# Supplementary material for: A Likelihood Approach for Real-Time Calibration of Stochastic Compartmental Epidemic Models
Source: PLoS Comput Biol. 2017 Jan 17;13(1):e1005257. doi: 10.1371/journal.pcbi.1005257 (PMC5240920; doi:10.1371/journal.pcbi.1005257)
Supplement: S1 File — (TAR.GZ) [file pcbi.1005257.s014.tar.gz › HSPH_Online-SI-Revision/Additions/EnKF08/n100-extreme-SEITR_noisy/n100-extreme-SEITR_noisy_EnKF_table4.pdf]

1	R0	{1.38654, 9.85565}	{2.3268, 5.67937}	{{1.43264, 2.20219}, {3., 7.22118}}	{67.8132, 42.3745}	inf	602.	12453.5	{599.051, 19183.6}	195.055	0.454336	newly	2726.36	11908.	{622., 21443.}	365.528	0.57732	Reff	0.128602	0.612987	{0.151914, 1.43229}	382.984	ar	92379.	206497.	{169640., 274357.}	123.532
2	R0	{2.36629, 12.2029}	{2.55591, 5.58973}	{{2.00749, 0.566543}, {3., 16.4485}}	{20.6687, 70.2038}	inf	104.	10132.7	{2367.64, 33325.9}	105.436	0.292238	newly	2664.	21734.3	{2020., 85389.}	732.604	0.603295	Reff	0.0301229	0.725698	{0.221977, 1.80974}	2312.69	ar	99022.	222541.	{190569., 284172.}	124.739
3	R0	{2.32253, 12.836}	{2.01569, 8.39328}	{{0.54166, 3.70232}, {3., 18.89}}	{46.4645, 52.1925}	inf	185.	5644.17	{838.997, 6961.96}	82.7703	0.49331	newly	3413.	4372.33	{582., 7294.}	104.412	0.393807	Reff	0.035767	0.281696	{0.0520849, 0.556551}	689.4	ar	98997.	192555.	{182459., 203360.}	94.5058
4	R0	{1.79075, 11.2058}	{2.61209, 6.06289}	{{2.01538, 0.578161}, {3., 17.476}}	{45.866, 69.3805}	inf	424.	9372.12	{2275.62, 36094.4}	85.2614	0.272253	newly	3304.	13654.9	{1941., 31172.}	338.032	0.449327	Reff	0.0739401	0.529314	{0.0902988, 1.14081}	623.742	ar	96920.	186663.	{163865., 245898.}	92.5946
5	R0	{2.26952, 9.62371}	{2.06197, 9.86011}	{{0.236087, 3.72959}, {3., 19.3234}}	{51.3716, 54.5921}	inf	39.	4191.76	{1509.67, 4910.78}	96.3615	0.195647	newly	1318.	3639.59	{598., 6158.}	205.242	0.318742	Reff	0.0289591	0.363082	{0.0340507, 0.53441}	1166.79	ar	98881.	178956.	{167983., 184897.}	80.9809
6	R0	{1.57191, 6.89004}	{2.51221, 7.48203}	{{1.83861, 4.24269}, {3., 11.5035}}	{59.8192, 40.0987}	inf	100.	6961.8	{1701.07, 10836.}	390.843	0.550339	newly	1057.	6853.36	{1440., 13020.}	558.471	0.595653	Reff	0.0825567	0.584942	{0.157967, 1.4787}	611.042	ar	95046.	194509.	{171339., 258592.}	104.647
7	R0	{1.99226, 17.2339}	{2.73535, 5.07248}	{{2.17374, 2.94179}, {3., 6.52404}}	{37.2988, 70.5669}	inf	602.	10885.1	{7632.89, 24120.3}	54.1314	0.249871	newly	3977.	13704.9	{5103., 37484.}	250.671	0.404699	Reff	0.0686533	0.617504	{0.105984, 1.33916}	807.33	ar	97992.	191479.	{170357., 237671.}	95.4031
8	R0	{2.63369, 15.1879}	{2.59741, 6.45275}	{{1.82445, 0.526787}, {3., 17.0459}}	{19.5163, 63.3751}	inf	100.	9885.73	{4112.98, 12640.}	87.6089	0.306186	newly	3068.	13847.6	{1778., 45447.}	371.517	0.434379	Reff	0.022123	0.56954	{0.10525, 1.32078}	2476.39	ar	99451.	201151.	{177863., 276557.}	102.261
9	R0	{2.53271, 9.90552}	{2.14556, 6.80337}	{{0.148872, 3.76453}, {3., 7.3861}}	{39.7816, 46.1935}	inf	27.	3139.2	{1456.46, 2983.17}	38.0907	0.157581	newly	1330.	2594.88	{984., 3561.}	105.8	0.300828	Reff	0.0191979	0.392061	{0.0287832, 0.629851}	1942.44	ar	99355.	175814.	{168874., 178293.}	76.9555
10	R0	{2.48791, 18.9003}	{2.14487, 7.23429}	{{0.509164, 5.51689}, {3., 7.87625}}	{40.1347, 61.724}	inf	387.	5746.1	{2062.48, 13045.2}	76.4641	0.672566	newly	4707.	4473.59	{1499., 9892.}	82.9783	0.404534	Reff	0.0381148	0.46192	{0.0640594, 0.758491}	1114.47	ar	99291.	176903.	{163270., 213513.}	78.1667
11	R0	{2.84414, 18.4765}	{2.53699, 6.99887}	{{2.15416, 3.66547}, {3., 8.30245}}	{18.0329, 62.1201}	inf	161.	6607.21	{404.488, 9276.97}	89.9431	0.794585	newly	4255.	5470.78	{374., 11596.}	112.482	0.490958	Reff	0.0214164	0.78437	{0.0910756, 1.45106}	3565.87	ar	99654.	185412.	{162170., 255424.}	86.0558
12	R0	{2.29369, 14.622}	{1.99472, 3.59582}	{{0.600457, 2.83381}, {3., 5.71487}}	{48.7552, 75.4081}	inf	189.	11669.9	{1601.35, 42854.8}	107.464	0.359449	newly	3139.	11801.7	{1784., 49892.}	298.346	0.446485	Reff	0.0347723	0.276299	{0.0864131, 0.454027}	696.834	ar	98982.	209241.	{183481., 283331.}	111.393
13	R0	{2.73413, 16.7529}	{2.46362, 4.75321}	{{1.21136, 2.03213}, {3., 5.98293}}	{21.7572, 71.6275}	inf	128.	4598.25	{725.18, 6847.36}	75.1394	0.602627	newly	3594.	5649.37	{693., 9679.}	116.542	0.361874	Reff	0.0212989	0.457734	{0.158555, 0.75942}	2049.1	ar	99543.	190777.	{177791., 206059.}	91.6528
14	R0	{1.79554, 16.5943}	{2.52957, 5.09432}	{{1.73469, 1.86608}, {3., 12.4047}}	{42.3016, 69.3008}	inf	1182.	17328.1	{3825.26, 40087.7}	78.2121	0.291071	newly	4850.	18775.6	{2230., 39922.}	304.314	0.540781	Reff	0.106063	0.511242	{0.192875, 0.77516}	390.038	ar	96821.	219277.	{185543., 290908.}	126.477
15	R0	{1.91429, 13.7771}	{2.49387, 4.86655}	{{1.3625, 3.12862}, {3., 6.29466}}	{47.9919, 64.6766}	inf	383.	7288.48	{4853.07, 11639.3}	52.2375	0.217013	newly	3380.	9628.95	{3390., 16045.}	185.113	0.291728	Reff	0.0667896	0.56674	{0.0871279, 0.981297}	752.485	ar	97541.	187270.	{168591., 203260.}	91.9906
16	R0	{1.42094, 10.5871}	{2.63274, 1.60472}	{{1.81072, 0.376745}, {3., 3.48283}}	{85.2813, 84.8427}	inf	1054.	4654.45	{1737.92, 7132.75}	47.0306	0.307266	newly	4015.	18712.2	{3458., 86191.}	374.329	0.434423	Reff	0.134932	0.898251	{0.539194, 1.81469}	566.878	ar	93044.	179466.	{155796., 240270.}	92.8833
17	R0	{1.45515, 6.16466}	{1.77084, 10.6681}	{{0.202729, 6.76672}, {3., 18.5532}}	{96.123, 73.8381}	inf	170.	4547.22	{1220.44, 3402.33}	172.154	0.34235	newly	1376.	2662.66	{430., 2961.}	120.831	0.317681	Reff	0.105091	0.203814	{0.0194494, 0.469693}	158.602	ar	93254.	185544.	{177032., 184268.}	98.9666
18	R0	{2.85669, 13.2416}	{2.44741, 7.26222}	{{1.82427, 4.25803}, {3., 11.1078}}	{19.6448, 45.1561}	inf	31.	7985.33	{1846.48, 15978.4}	82.0295	0.321629	newly	2583.	7753.84	{1239., 17603.}	226.221	0.41119	Reff	0.014512	0.594795	{0.232115, 1.24199}	3999.62	ar	99644.	203787.	{183651., 259922.}	104.515
19	R0	{2.56417, 17.9558}	{2.49892, 9.88346}	{{1.85069, 7.58689}, {3., 10.998}}	{24.6784, 44.9567}	inf	266.	14441.	{983.494, 57005.4}	104.997	0.533173	newly	4524.	8730.4	{547., 27574.}	142.557	0.381004	Reff	0.0335137	0.542577	{0.141095, 1.0988}	1523.98	ar	99389.	176210.	{149475., 245670.}	77.2931
20	R0	{2.27517, 11.1864}	{2.53118, 6.59969}	{{1.87364, 4.20521}, {3., 11.6183}}	{25.7242, 42.856}	inf	92.	6324.91	{1638.91, 10375.}	86.3996	0.251398	newly	2013.	6750.19	{1360., 12013.}	250.657	0.355244	Reff	0.0313746	0.566333	{0.159644, 1.20274}	1710.47	ar	98894.	193622.	{175546., 254447.}	95.7871
21	R0	{2.35735, 10.4735}	{1.8173, 10.7683}	{{0.191404, 3.72983}, {3., 19.3692}}	{53.9876, 52.1458}	inf	65.	4108.94	{1259.67, 5748.94}	70.4124	0.27023	newly	1848.	2425.4	{484., 3492.}	71.8128	0.261244	Reff	0.0275103	0.358992	{0.0316726, 0.661678}	1205.02	ar	99036.	183517.	{174186., 207535.}	85.3035
22	R0	{1.79551, 11.9179}	{2.22588, 3.65453}	{{0.636256, 2.84076}, {3., 5.34154}}	{63.0517, 69.3358}	inf	355.	10729.	{2410.48, 32025.}	102.682	0.271908	newly	2882.	11733.2	{2635., 25391.}	314.823	0.473569	Reff	0.0710663	0.357412	{0.066496, 0.646457}	413.342	ar	97033.	197348.	{170123., 260682.}	103.382
23	R0	{1.23545, 6.31687}	{2.53265, 5.70895}	{{1.43974, 3.34817}, {3., 6.76292}}	{105.296, 18.5289}	inf	264.	8576.38	{4334.36, 6783.45}	419.316	0.611237	newly	1370.	7789.41	{3900., 9193.}	470.277	0.670842	Reff	0.148353	0.581741	{0.285794, 0.928818}	293.014	ar	88739.	199995.	{183778., 228561.}	125.375
24	R0	{2.24089, 10.1789}	{2.44043, 6.81764}	{{1.80943, 4.27391}, {3., 11.4043}}	{27.0033, 39.5227}	inf	88.	7324.45	{1774.31, 6579.95}	105.132	0.265277	newly	2182.	7941.41	{1696., 9408.}	278.124	0.375401	Reff	0.0325825	0.602986	{0.248863, 1.32657}	1751.58	ar	98868.	192203.	{171440., 250831.}	94.4037
25	R0	{1.57298, 10.2607}	{2.67316, 7.76554}	{{2.13989, 3.97348}, {3., 9.91698}}	{69.9424, 24.3177}	inf	633.	5971.03	{601.023, 15905.6}	100.927	0.572565	newly	3489.	6621.	{473., 18016.}	176.157	0.515752	Reff	0.10572	0.724217	{0.252407, 1.64794}	585.033	ar	94957.	174103.	{151187., 256293.}	83.3488
26	R0	{1.2154, 8.2576}	{2.48451, 3.24595}	{{1.14467, 2.76843}, {3., 4.07313}}	{107.204, 60.6913}	inf	684.	3416.44	{1143.65, 2232.98}	81.8029	0.350088	newly	2648.	4189.93	{1410., 3594.}	81.0434	0.16612	Reff	0.165416	0.484879	{0.265135, 0.650353}	194.167	ar	88421.	174216.	{160477., 210219.}	97.0298
27	R0	{2.22841, 18.7983}	{2.43792, 6.37228}	{{1.06782, 3.37848}, {3., 11.7083}}	{35.3822, 66.1018}	inf	454.	6686.26	{783.624, 12631.}	89.6035	0.747198	newly	4125.	5279.72	{752., 13312.}	104.063	0.426786	Reff	0.0518997	0.580616	{0.22475, 0.845546}	1026.71	ar	98756.	182714.	{163991., 234964.}	85.0158
28	R0	{1.27778, 5.36767}	{1.83436, 11.3168}	{{0.210942, 6.97783}, {3., 19.0884}}	{112.746, 116.322}	inf	160.	6515.74	{1195.37, 16689.8}	494.745	0.534213	newly	965.	3763.91	{463., 11336.}	310.262	0.428337	Reff	0.136275	0.316837	{0.0365143, 0.55018}	181.366	ar	89843.	192539.	{177091., 243166.}	114.306
29	R0	{2.38042, 15.8901}	{2.28022, 3.48061}	{{1.18376, 3.23903}, {3., 4.06175}}	{32.3199, 78.0958}	inf	266.	13034.3	{1675.26, 42762.8}	84.5282	0.378715	newly	4278.	15944.4	{2597., 45128.}	289.781	0.45893	Reff	0.0399673	0.508529	{0.136032, 0.913056}	1177.96	ar	99112.	187992.	{159727., 255950.}	89.6764
30	R0	{2.53188, 12.7607}	{1.97449, 9.41646}	{{0.465396, 3.75807}, {3., 19.4362}}	{43.462, 52.0535}	inf	140.	6635.68	{2404.22, 12172.4}	62.437	0.325444	newly	3519.	4096.21	{1037., 8524.}	68.7999	0.270985	Reff	0.0262556	0.288148	{0.0395462, 0.490421}	1000.86	ar	99317.	183491.	{172026., 195901.}	84.7524
31	R0	{1.24482, 10.5471}	{2.09332, 5.12998}	{{0.391767, 1.14931}, {3., 7.59998}}	{115.136, 51.3612}	inf	1178.	16363.9	{3632.37, 57208.7}	185.483	0.316399	newly	3288.	16118.3	{3104., 46987.}	396.122	0.530881	Reff	0.174511	0.536688	{0.132169, 1.01915}	221.129	ar	89097.	206936.	{179500., 279518.}	132.259
32	R0	{2.30446, 17.9607}	{2.00827, 4.838}	{{0.601856, 1.787}, {3., 11.8628}}	{48.4679, 73.0634}	inf	386.	14134.7	{3360.2, 51089.3}	89.676	0.374815	newly	4308.	13786.6	{3231., 46126.}	235.214	0.391754	Reff	0.0453748	0.303171	{0.0848366, 0.627602}	568.574	ar	99028.	189005.	{164481., 279271.}	90.86
33	R0	{2.31579, 11.8236}	{1.83306, 2.61107}	{{0.239049, 0.954964}, {3., 3.69473}}	{54.5269, 77.9164}	inf	79.	6496.92	{374.31, 12940.3}	92.4563	0.334559	newly	2012.	7402.3	{395., 18007.}	296.04	0.524638	Reff	0.0280905	0.267908	{0.0341905, 0.463689}	860.422	ar	99012.	180635.	{165236., 220118.}	82.4377
34	R0	{1.6678, 10.6744}	{1.87791, 4.43938}	{{0.589034, 2.32098}, {3., 6.51988}}	{72.2341, 58.411}	inf	325.	14143.4	{1723.34, 51161.6}	228.16	0.430314	newly	2446.	13933.2	{1521., 43833.}	485.294	0.614729	Reff	0.0850078	0.368383	{0.108553, 0.704952}	342.994	ar	95809.	202124.	{170873., 290724.}	110.966
35	R0	{1.65476, 7.07559}	{1.95366, 11.6368}	{{0.171948, 7.05467}, {3., 18.6098}}	{82.2851, 67.6134}	inf	106.	5275.66	{1342.91, 9205.71}	222.673	0.382598	newly	1151.	2953.38	{443., 3511.}	179.536	0.375362	Reff	0.0754074	0.307982	{0.0287877, 0.538068}	348.662	ar	95746.	185439.	{175399., 190784.}	93.678
36	R0	{2.71807, 18.5959}	{2.10577, 7.1208}	{{0.731419, 5.4837}, {3., 7.78212}}	{34.9741, 61.7077}	inf	279.	4509.34	{433.915, 9664.8}	85.9749	0.911045	newly	5029.	3546.9	{294., 6817.}	92.3504	0.61741	Reff	0.0291921	0.445462	{0.154742, 0.776905}	1426.92	ar	99540.	167075.	{151076., 204595.}	67.8469
37	R0	{2.6202, 12.7552}	{2.42058, 3.61228}	{{1.30553, 3.25474}, {3., 4.73274}}	{25.0126, 71.68}	inf	74.	14533.1	{2148.27, 33600.9}	141.234	0.36989	newly	2840.	22401.8	{2490., 75833.}	698.249	0.732249	Reff	0.0220097	0.698757	{0.162461, 1.53422}	3075.4	ar	99426.	219437.	{183002., 302890.}	120.704
38	R0	{1.47672, 9.81651}	{2.56924, 9.74603}	{{1.70776, 5.84339}, {3., 12.1834}}	{73.9831, 22.4837}	inf	503.	4886.48	{521.026, 10639.3}	100.174	0.508556	newly	2643.	3317.01	{281., 7251.}	108.802	0.491823	Reff	0.109425	0.686287	{0.16504, 1.47444}	537.794	ar	93815.	178284.	{156911., 230904.}	90.0383
39	R0	{1.90004, 12.3313}	{2.1981, 7.02855}	{{0.344638, 5.43118}, {3., 7.65291}}	{62.3536, 43.0023}	inf	369.	3072.5	{790.164, 3642.74}	73.7182	0.562849	newly	3317.	2353.47	{648., 2773.}	66.5164	0.376754	Reff	0.0681354	0.425006	{0.0578971, 0.598949}	534.8	ar	97413.	165433.	{158235., 174589.}	69.8259
40	R0	{2.64218, 18.5237}	{2.14392, 6.05336}	{{0.898437, 3.52217}, {3., 7.539}}	{34.7523, 67.321}	inf	174.	14799.3	{1280.3, 45307.}	97.2449	0.401859	newly	3674.	14163.5	{1795., 48657.}	308.394	0.446518	Reff	0.0267917	0.424121	{0.0548231, 0.901272}	1484.99	ar	99474.	203466.	{175129., 265615.}	104.542
41	R0	{1.6945, 7.17996}	{1.92823, 11.9083}	{{0.350956, 6.87665}, {3., 18.5864}}	{74.0361, 70.4174}	inf	141.	4963.1	{1711.28, 8018.42}	112.44	0.287833	newly	1635.	2766.66	{616., 3226.}	97.7823	0.296455	Reff	0.0694067	0.341399	{0.0605915, 0.534796}	401.267	ar	96332.	182347.	{172392., 205925.}	89.2901
42	R0	{1.84559, 18.0074}	{2.3554, 4.36044}	{{1.15274, 2.55038}, {3., 5.83003}}	{53.6916, 75.7853}	inf	1048.	5161.13	{947.647, 7254.66}	79.8309	0.687783	newly	4650.	8202.39	{2261., 18179.}	131.359	0.37231	Reff	0.093313	0.746689	{0.0718768, 1.56679}	713.313	ar	97358.	190810.	{170635., 214711.}	95.9878
43	R0	{2.24965, 17.0361}	{2.25367, 9.00277}	{{0.602746, 6.2921}, {3., 10.9595}}	{45.1828, 47.1548}	inf	520.	8307.11	{2837.28, 19289.}	73.5384	0.502716	newly	4598.	5134.03	{1445., 12302.}	78.9933	0.338349	Reff	0.0537891	0.394365	{0.0462188, 0.725667}	640.361	ar	98770.	184129.	{165855., 220863.}	86.4224
44	R0	{2.54324, 15.7967}	{2.20102, 3.4367}	{{0.260501, 2.7323}, {3., 5.04942}}	{36.4446, 78.2442}	inf	127.	8254.05	{1291.29, 26950.5}	87.1587	0.459826	newly	3246.	10313.	{1514., 26783.}	250.58	0.410261	Reff	0.0255087	0.374323	{0.0487158, 0.617586}	1373.	ar	99354.	185663.	{163374., 260797.}	86.8698
45	R0	{2.54943, 14.8032}	{2.28701, 7.46261}	{{1.20677, 1.07453}, {3., 17.7542}}	{30.7231, 62.3126}	inf	120.	11348.5	{1626.63, 23329.6}	96.9643	0.338334	newly	3187.	15063.	{1091., 29532.}	412.238	0.545472	Reff	0.0250609	0.523476	{0.0666094, 0.892585}	1989.46	ar	99380.	197032.	{167683., 262667.}	98.2612
46	R0	{2.37755, 12.6461}	{1.77676, 9.11909}	{{0.447439, 3.73681}, {3., 19.5485}}	{50.9261, 51.1511}	inf	129.	6406.21	{2537.49, 9675.6}	74.2511	0.357618	newly	3124.	4796.65	{992., 8934.}	110.556	0.306056	Reff	0.0309795	0.278862	{0.0652506, 0.511049}	805.237	ar	99096.	175648.	{161588., 231192.}	77.2504
47	R0	{1.69279, 11.6636}	{2.50436, 7.02785}	{{0.639937, 3.56614}, {3., 8.68165}}	{64.8122, 39.7454}	inf	535.	1742.12	{258.81, 2593.39}	79.3739	0.86009	newly	3657.	1822.68	{189., 3631.}	79.3426	0.67579	Reff	0.0899041	0.557351	{0.091498, 1.12437}	532.879	ar	96214.	168579.	{158973., 183666.}	75.2124
48	R0	{1.82402, 10.0038}	{2.52699, 9.92116}	{{2.02385, 7.04946}, {3., 17.9735}}	{42.2709, 42.2199}	inf	312.	11218.	{3450.72, 11612.3}	130.673	0.250766	newly	2763.	7264.58	{1715., 13443.}	180.01	0.345415	Reff	0.0676164	0.606001	{0.115047, 1.46849}	805.282	ar	97076.	216875.	{190364., 277353.}	123.407
49	R0	{1.6424, 11.1667}	{2.10774, 6.49539}	{{0.235138, 0.440777}, {3., 10.0381}}	{79.2119, 41.8425}	inf	368.	8207.08	{1213.31, 11717.3}	140.363	0.379312	newly	2454.	6244.2	{836., 18159.}	202.986	0.379591	Reff	0.0891166	0.345448	{0.0384751, 0.49039}	307.134	ar	95546.	184445.	{167607., 202442.}	93.0433
50	R0	{2.05638, 14.3048}	{2.05314, 5.75163}	{{0.907529, 3.52763}, {3., 6.93363}}	{49.2979, 59.7923}	inf	362.	10316.2	{4214.18, 15346.8}	84.1887	0.297842	newly	3752.	8768.62	{3759., 20209.}	139.707	0.236356	Reff	0.0564271	0.442934	{0.162934, 0.71155}	687.143	ar	98194.	197957.	{180813., 220790.}	101.598
